# Bioactive Peptide‐Enriched Requeson: A Functional Dairy Product From Whey Fermentation

**DOI:** 10.1002/fsn3.71806

**Published:** 2026-04-17

**Authors:** Luis‐Fernando Patlan‐Velázquez, Luis‐Guillermo González‐Olivares, Mariano García‐Garibay, Sergio Alatorre‐Santamaría, Lorena Gómez‐Ruiz, Gabriela Rodríguez‐Serrano, Ulises Carrasco‐Navarro, Alma Cruz‐Guerrero

**Affiliations:** ^1^ Departamento de Biotecnología Universidad Autónoma Metropolitana‐Iztapalapa Ciudad de Mexico; ^2^ Área Académica de Química Universidad Autónoma del Estado de Hidalgo Hidalgo Mexico; ^3^ Departamento de Ciencias de la Alimentación Universidad Autónoma Metropolitana‐Lerma Lerma de Villada Mexico

**Keywords:** bioactive peptides, co‐culture, functional food, lactic acid bacteria, requeson cheese, whey

## Abstract

Requeson, a traditional Mexican whey cheese, represents a promising avenue for the valorization of dairy by‐products. However, its functional potential remains underexplored. This study evaluated the enhancement of bioactive properties in Requeson cheese through lactic acid bacteria (LAB) fermentation, comparing two monocultures ( *Lactobacillus delbrueckii* subsp. *bulgaricus* NCFB 2772 and *Streptococcus thermophilus* SY‐102) and a co‐culture of both strains, cultured in sweet whey. Fermented whey was used to produce Requeson cheese, and peptide‐rich extracts were analyzed for antioxidant activity (DPPH and FRAP), mineral‐chelating capacity (Fe^2+^, Ca^2+^, Mg^2+^), enzymatic inhibition (ACE‐I, DPP‐IV), and antimicrobial activity against *E. coli* , *M. luteus* , and *L. innocua* . Liquid chromatography–tandem mass spectrometry (LC–MS/MS) was employed to identify peptides, and their potential bioactivities were predicted using the UniDL4BioPep platform. The co‐culture fermentation yielded significantly higher values across all assays, including antioxidant capacity (41.3 mg Trolox), ACE‐I inhibition (31.8%), DPP‐IV inhibition (14.2%), and antimicrobial activity. Several peptides with predicted antioxidant, antihypertensive, antidiabetic, and antimicrobial properties were exclusive to the co‐culture formulation. These findings suggest that LAB co‐culture fermentation of sweet whey is an effective strategy to develop peptide‐enriched functional Requeson cheese with health‐promoting properties. This approach supports the sustainable use of dairy by‐products and contributes to the advancement of functional dairy products in the bioeconomy.

## Graphical abstract

This study presents the production of functional Requeson from whey using lactic fermentation. Proteolysis induced by *Lactobacillus delbrueckii* subsp. *bulgaricus* and *Streptococcus thermophilus* promotes the generation of bioactive peptides. The product is evaluated for antioxidant, ACE‐inhibitory, DPP‐IV inhibitory, mineral chelating, and antimicrobial activities. LC–MS/MS analysis enables peptide identification and bioactivity prediction, highlighting whey valorization into a functional dairy product.

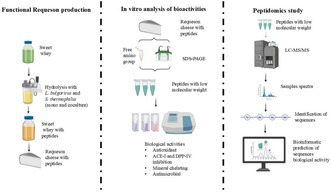

## 1 Introduction

Bioactive peptides are protein fragments that positively impact human health and have gained increasing attention due to their potential role in preventing chronic diseases such as diabetes and cardiovascular conditions (Lemes et al. 2023). These peptides are embedded in proteins from both animal and plant sources and are released through various hydrolytic mechanisms, including enzymatic digestion, chemical treatment, and microbial proteolysis (Akbarian et al. 2022).

Whey, a major by‐product of the cheese industry, is a rich source of such bioactive peptides. Despite representing nearly 90% of the volume of milk used during cheese manufacturing, whey is often discarded or underutilized, contributing to environmental pollution or being relegated to animal feed (Sebastián‐Nicolás et al. 2020). Due to its high protein content, primarily β‐lactoglobulin and α‐lactalbumin, whey has been extensively explored as a substrate for the production of bioactive peptides. These peptides, generated through enzymatic or microbial hydrolysis, have demonstrated diverse biological activities, including antioxidant, antihypertensive, antidiabetic, and antimicrobial effects (Ungureanu‐Rusu et al. 2025).

Various methods have been developed to obtain these compounds from whey proteins, ranging from enzymatic hydrolysis using specific proteases to non‐enzymatic approaches such as ultrasound‐assisted extraction (Gallego et al. 2024). However, microbial proteolysis has gained relevance in recent years, as this technique generates less waste and fewer undesirable secondary compounds that require further purification. This enables in situ application within food matrices, supporting the development of functional foods (Chai et al. 2020).

Several factors influence peptide generation via microbial proteolysis, including the carbon and nitrogen sources, the growth conditions of the microorganism, and its proteolytic system. This is particularly important, as peptide yield and functionality can vary depending on whether monocultures or microbial co‐cultures are employed (Olvera‐Rosales, Pérez‐Escalante, et al. 2023; Guzmán‐Rodríguez et al. 2024).

Whey cheeses represent one of the principal applications of discarded whey. Their production involves protein precipitation induced by heat treatment and the addition of organic acids, with optional ingredients (e.g., salts, milk, cream, or spices) incorporated according to regional practices (Bintsis and Papademas 2023). In Mexico, a major application of whey is the production of Requeson, a fresh cheese obtained through thermal and acid‐induced precipitation of whey proteins, followed by curd separation. This product is widely consumed due to its relatively high protein content (approximately 12%) and low production cost, as whey constitutes its sole raw material, unlike Ricotta and other whey cheeses (Mazorra‐Manzano et al. 2025).

Although numerous studies have investigated the production of bioactive peptides from whey using proteolytic bacterial strains, limited attention has been devoted to incorporating peptide‐enriched whey into Requeson production (Solieri et al. 2022). Therefore, the present study aimed to develop and characterize a functional Requeson cheese with bioactive peptides. Sweet whey was fermented using lactic acid bacteria under three distinct systems: two monocultures ( *Lactobacillus delbrueckii* subsp. *bulgaricus* NCFB 2772 or *Streptococcus thermophilus* SY‐102) and a co‐culture combining both strains. The resulting products were evaluated in vitro for their biological activities, including antioxidant activity, mineral‐chelating capacity, angiotensin‐converting enzyme (ACE‐I) inhibition, dipeptidyl peptidase‐IV (DPP‐IV) inhibition, and antimicrobial activity. Bioactive peptides were further characterized to assess their potential contribution to the observed functional properties. These findings may support the development of fermented sweet whey–based Requeson as a functional dairy product with potential health‐related benefits.

## 2 Materials and Methods

### 2.1 Bacteria, Chemicals and Reagents

*Lactobacillus delbrueckii* subsp. *bulgaricus* NCFB 2772, *Streptococcus thermophilus* SY‐102, *Escherichia coli* K12, *Micrococcus luteus* CD‐BBB1018, and *Listeria innocua* ATCC 33090 were obtained from the bacterial culture collection of the food biotechnology laboratory of the Universidad Autónoma Metropolitana, Campus Iztapalapa (Mexico City, Mexico). Man, Rogosa, and Sharp (MRS) broth and agar, Skim Milk medium, nutrient broth, brain heart infusion and casein peptone were obtained from Difco Company (Detroit, Michigan, USA). 2,2‐diphenyl‐l‐picrylhydrazyl (DPPH), Trolox, 2,4,6‐Tri (2‐pyridyl)‐s‐triazine (TPTZ), sodium acetate, angiotensin I‐converting enzyme (ACE‐I, EC 3.4.15.1), Hypuryl‐His‐Leu, sodium borate, sodium chloride, pyridine, benzenesulfonyl chloride, dipeptidyl peptidase IV enzyme (DPP‐IV, D4943‐1VL), Gly‐Pro‐pNA, potassium carbonate, ferrozine, hydroxylamine, picryl sulfonic acid and Coomassie blue G‐250 were obtained from Sigma‐Aldrich Co (St. Louis, USA). Hydrochloric, phosphoric, trichloroacetic and glacial acetic acids, methanol and absolute ethanol, KH_2_PO_4_, NaOH, FeCl_3_.6H_2_O, FeSO_4_.7H_2_O, FeCl_2_, CaCl_2_.2H_2_O and MgCl_2_.6H_2_O were obtained from J.T. Baker (NJ, USA). Bradford 1× dye reagent, glycine, tricine, sodium dodecyl sulfate (SDS), tetramethylethylenediamine (TEMED), ammonium persulfate and acrylamide solution (30%, 19:1 acrylamide:bisacrylamide ratio and 5% crosslinker) were purchased from BioRad, Hercules (CA, USA). Magnesium xylidyl and calcium arsenazo III were purchased from Spinreact (Naucalpan de Juárez, México). Rennet was purchased from CHR Hansen (Mexico City, Mexico). All reagents were of analytical grade.

### 2.2 Requeson Cheese Production

Given the significant variability in the native microbiota of commercial whey in Mexico, which has been reported to exhibit proteolytic activity (Mazorra‐Manzano et al. 2020), standardized sweet whey was produced from commercial milk (Lala) by adding liquid rennet (200 μL/L) and heating to 32°C until complete curd formation (40 min). The resulting whey was pasteurized (65°C, 30 min) and stored frozen until use (Parra‐Ocampo et al. 2020). The obtained whey exhibited a pH of 6.7, corresponding to sweet whey (pH > 6.5), which is suitable for controlled lactic fermentation.

Three fermentation systems were evaluated to determine the most effective strategy for bioactive peptide production: a monoculture of *Lactobacillus delbrueckii* subsp. *bulgaricus* NCFB 2772, a monoculture of *Streptococcus thermophilus* SY‐102, and a co‐culture combining both strains. These strains were selected based on their Generally Recognized as Safe (GRAS) status and their previously reported capacity to generate bioactive peptides (Sebastián‐Nicolas et al. 2021; Guzmán‐Rodríguez et al. 2024).

Each strain was sequentially propagated in MRS broth, skim milk, and finally in pasteurized sweet whey until reaching a cell density of 1 × 10^7^ CFU/mL, which was used as the starter culture. For monoculture fermentations, pasteurized sweet whey was inoculated with 5% (v/v) of the respective starter culture. For the co‐culture system, 2.5% (v/v) of each strain was added to achieve an equivalent total inoculum volume. All fermentations were incubated at 37°C. The process was terminated when the pH reached 5.0 ± 0.2, a value reported as suitable for subsequent Requeson production (Ramírez‐Rivas et al. 2022).

After fermentation, the whey was heated at 90°C for 1 h to induce protein precipitation. The coagulated material was filtered through sterile cotton cloth and allowed to drain overnight at 4°C to separate the secondary whey. The recovered Requeson was transferred into sterile containers and stored at 4°C until analysis. Treatments were designated as LB ( *L. bulgaricus* ), ST ( *S. thermophilus* ), and CC (*co‐culture*).

### 2.3 Proteolytic Analysis

Bacterial proteolytic activity was assessed by quantifying free amino groups as an indirect indicator of proteolysis, and by profiling low‐molecular‐weight peptides. Two analytical approaches were employed: the trinitrobenzenesulfonic acid (TNBS) assay for quantification of free amino groups and Tris–Tricine SDS‐PAGE for peptide profiling.

For sample preparation, 30 g of each Requeson sample were homogenized in 90 mL of 50 mM phosphate buffer (pH 7.0) for 1 min and incubated at 40°C for 1 h under agitation (150 rpm). Trichloroacetic acid (TCA, 80% w/v) was then added at a 1:6 (v/v; TCA:sample) ratio to precipitate high‐molecular‐weight proteins. Samples were centrifuged at 15,000 ***g*** for 30 min at 4°C, and the supernatant was collected and stored at −20°C until analysis. Total protein concentration was determined using the Bradford method (Bradford 1976).

Free amino groups were quantified following an adapted TNBS method (Sebastián‐Nicolas et al. 2021). Briefly, 250 μL of supernatant were mixed with 2 mL of 0.2 M phosphate buffer (pH 8.2) and 2 mL of 0.1% (w/v) TNBS solution in light‐protected tubes. The mixture was incubated at 50°C for 60 min, and the reaction was stopped by adding 4 mL of 0.1 N HCl. Absorbance was measured at 340 nm. A blank containing 250 μL of deionized water was included. Quantification was performed using a glycine standard curve (50 to 250 μg/L), and results were expressed as μg glycine equivalents per gram of Requeson.

Peptide profiles were analyzed by Tris‐Tricine SDS‐PAGE as described by González‐Olivares et al. (2014) with minor modifications. Supernatants were lyophilized (Scientz‐10 N, Ningbo Scientz Biotechnology) and resuspended in 0.5 M Tris–HCl buffer (pH 6.8) to a final protein concentration of 300 μg/mL. Samples were mixed (2:1, v/v) with loading buffer containing 0.5 M Tris–HCl (pH 6.8), 1% (w/v) SDS, 4% (v/v) 2‐mercaptoethanol, 0.02% (w/v) Coomassie Brilliant Blue, and 24% (w/v) glycerol. After incubation at 40°C for 30 min, 20 μL were loaded onto a 16.5% resolving gel with a 4% stacking gel. Electrophoresis was conducted at 30 V through the stacking gel and 95 V during separation. Gels were stained using the Blue Silver method (Candiano et al. 2004) and documented using a Gel‐Doc imaging system (Bio‐Rad, USA).

### 2.4 Peptide Extraction

The supernatants obtained after TCA precipitation (Section 2.3) were subjected to ultrafiltration using 10 kDa molecular weight cut‐off centrifugal filters (Nanosep, Pall Corporation, Michigan, USA). The permeate fraction (< 10 kDa), corresponding to low‐molecular‐weight peptides, was collected and subsequently lyophilized (Scientz‐10 N, Ningbo Scientz Biotechnology).

For biological activity assays, lyophilized peptide extracts were reconstituted in deionized water at 15 mg/mL for antioxidant, angiotensin‐converting enzyme (ACE) inhibitory, and dipeptidyl peptidase‐IV (DPP‐IV) inhibitory assays. For mineral‐chelating activity, samples were reconstituted at 50 mg/mL in the corresponding mineral solutions as described below. For antimicrobial assays, peptide extracts were prepared at 5 mg/mL in sterile deionized water.

### 2.5 Antioxidant Activity

The antioxidant activity was assessed by two different methods: the 2,2‐diphenyl‐1‐picrylhydrazyl (DPPH) assay, which measures free radical scavenging capacity, and the Ferric Reducing Ability of Plasma (FRAP) assay, which assesses reducing power via electron transfer.

The DPPH radical scavenging activity was determined according to Sebastián‐Nicolás et al. (2024) with some adjustments. Briefly, 100 μL of the sample was mixed with 2.9 mL of 0.1 mM DPPH solution in methanol in light‐protected tubes. The reaction mixture was incubated in the dark for 50 min at room temperature, and the absorbance (A) was measured at 515 nm (using a UV–Vis spectrophotometer (Shimadzu UV‐1800‐120 V)). Following this, the absorbance of absolute methanol (B) and a control consisting of 100 μL of methanol mixed with 2.9 mL of DPPH solution (C) were prepared under the same conditions. The percentage of remaining DPPH was calculated using Equation (1).

(1) $$\text{DPPH remaining}\;\left(\%\right)=\left[\left(A-B\right)/C\right]\times 100$$

A calibration curve was generated by plotting Trolox concentration versus the percentage of remaining DPPH, and the antioxidant capacity of the samples was expressed as milligrams of Trolox.

Ferric reducing antioxidant power (FRAP) was determined according to Kotsaki et al. (2025), with minor modifications. The FRAP reagent was freshly prepared by mixing 300 mM acetate buffer (pH 3.6), 10 mM TPTZ solution in 40 mM HCl, and 20 mM FeCl₃ in a 10:1:1 (v/v/v) ratio. For analysis, 8.75 mL deionized water, 1 mL FRAP reagent, and 250 μL of sample were combined in light‐protected tubes and incubated at 37°C for 10 min. Absorbance was measured at 593 nm against a reagent blank. The results for each sample were expressed as milligrams of Fe^2+^.

### 2.6 Mineral Chelating Activity

Mineral‐chelating activity was determined according to González‐Olivares et al. (2023), with minor modifications. All glassware and materials were pretreated by immersion in 1 N HCl for 24 h, rinsed thoroughly with deionized water, and dried at 95°C to eliminate residual metal contamination.

For iron chelation, the sample was incubated with an Fe^2+^ solution (15 mg/L). An aliquot (500 μL) of this mixture was combined with 100 μL of 1.4 M hydroxylamine hydrochloride and incubated for 10 min. Subsequently, 100 μL of 5 mM ferrozine was added to complex residual Fe^2+^. Absorbance was measured at 562 nm.

For calcium chelation, the sample was incubated with a Ca^2+^ solution (800 mg/L). And aliquot (50 μL) was diluted 1:10 in 0.2 M phosphate buffer (pH 8.0), and 10 μL of the diluted solution were mixed with 1 mL of calcium arsenazo III reagent. Absorbance was measured at 650 nm.

For magnesium chelation, the sample was incubated in an Mg^2+^ solution (350 mg/L). And aliquot (50 μL) was diluted 1:10 in 0.4 M Britton–Robinson buffer (pH 11.2), and 10 μL were mixed with 1 mL of magnesium xylidyl blue reagent before measurement of absorbance at 546 nm.

For each assay, absorbance was recorded immediately after reagent addition (T1) and after 1 h of incubation (T2). Chelating activity was calculated using Equation (2):

(2) $$\text{Chelating activity}\;\left(\%\right)=\left[\left(\text{T1}-T2\right)/\text{T1}\right]\times 100$$

### 2.7 Angiotensin Converting Enzyme (ACE‐I) Inhibitory Activity

ACE‐I inhibitory activity was determined according to Hernández‐Riveros et al. (2024), with some modifications. Briefly, an aliquot (80 μL) of sample was mixed with 200 μL of substrate solution (5 mM hippuryl‐histidyl‐leucine in 0.1 M sodium borate buffer, pH 8.3) and 20 μL of ACE‐I solution (0.1 U/mL). The reaction mixture was incubated at 37°C for 80 min. The reaction was terminated by adding 250 μL of 0.1 N HCl. Subsequently, 1.7 mL of ethyl acetate was added, and the mixture was vortexed three times to extract the liberated hippuric acid. After phase separation, 800 μL of the organic phase were collected and evaporated at 85°C for 1 h. The residue was resuspended in 500 μL of deionized water, followed by addition of 150 μL of benzenesulfonyl chloride and 300 μL of pyridine. Absorbance was measured at 410 nm (A_s_). A control reaction (A_100_) was performed by replacing the sample with 80 μL of borate buffer under identical conditions. ACE inhibitory activity (%) was calculated with Equation (3):

(3) $$\mathrm{ACE}\;\text{inhibition}\;\left(\%\right)=\left[\left(A_{100}-A_{s}\right)/A_{100}\right]\times 100$$

### 2.8 Inhibition of the Dipeptidyl Peptidase IV (DPP‐IV) Enzyme

DPP‐IV inhibitory activity was determined according to García‐Escamilla et al. (2024), with minor modifications. Briefly, an aliquot (100 μL) of sample solution was mixed with 100 μL of the substrate solution (1.6 mM Gly‐Pro‐pNA dissolved in 0.1 M Tris–HCl buffer, pH 8) and preincubated at 37°C for 10 min. Subsequently, 200 μL of DPP‐IV enzyme solution (0.01 U/mL) was added to initiate the reaction, which proceeded for 60 min at 37°C. The reaction was terminated by adding 400 μL of 0.1 M Na₂CO₃, and absorbance was measured at 405 nm (A). A positive control (B), consisting of 100 μL of 0.1 M Tris–HCl buffer (pH 8.0) instead of the sample, was prepared under identical conditions. A sample blank (C) containing 100 μL of sample solution, 300 μL of 0.1 M Tris–HCl buffer (pH 8.0), and 400 μL of 0.1 M Na₂CO₃ (without enzyme and substrate) was used to correct for background absorbance. The DPP‐IV inhibitory activity (%) was calculated using Equation (4):

(4) $$\mathrm{DPP}-\mathrm{IV}\;\text{inhibition}\;\left(\%\right)=\left[\left(B-\left(A-C\right)\right)/B\right]\times 100$$

### 2.9 Antimicrobial Activity

Antimicrobial activity was evaluated using a turbidimetric growth inhibition assay according to Innocente et al. (2023), with minor modifications. *Escherichia coli* (K12), *Micrococcus luteus* (CD‐BBB1018), and *Listeria innocua* (ATCC 33090) were used as indicator strains. Overnight cultures were grown at 37°C in their respective media: nutrient broth for *E. coli* , brain–heart infusion broth for *M. luteus* , and nutrient broth supplemented with glucose (2.5 g/L), NaCl (5 g/L), and casein peptone (15 g/L) for *L. innocua* .

Fresh cultures were prepared and incubated at 37°C with agitation (150 rpm) until reaching approximately 1 × 10^7^ CFU/mL, as estimated by optical density at 600 nm (OD_600_ < 0.12). An aliquot (2 mL) of each culture was mixed with 0.5 mL of peptide extract and incubated at 37°C for 3 h. Absorbance was measured at 600 nm (As). A growth control (Ac) consisted of 2 mL of culture without a peptide extract. Sterile medium served as a blank. Antimicrobial activity, expressed as percentage growth inhibition, was calculated using Equation (5):

(5) $$\text{Antimicrobial activity}\;\left(\%\right)=\left[\left(\mathrm{Ac}-\mathrm{As}\right)/\mathrm{Ac}\right]\times 100$$

### 2.10 Screening of Bioactive Peptides

All Requeson formulations were analyzed by Liquid Chromatography–Tandem Mass Spectrometry (LC–MS/MS) at the Institut de Recherches Cliniques de Montréal (IRCM), Canada. Prior to analysis, Requeson samples were prepared as described in Section 2.4. Lyophilized extracts were reconstituted in 1% (v/v) acetonitrile containing 1% (v/v) formic acid and loaded onto a self‐packed C18 analytical column coupled to an Easy‐nLC 1200 system (Proxeon Biosystems, Roskilde, Denmark).

Peptide separation was performed using mobile phase A (0.2% formic acid in water) and mobile phase B (90% acetonitrile with 0.2% formic acid) under a linear gradient at a flow rate of 250 nL/min. The LC system was interfaced with an Orbitrap Fusion Tribrid mass spectrometer (Thermo Scientific, Waltham, MA, USA) equipped with a Nanospray Flex ion source. Precursor ions were fragmented by higher‐energy collisional dissociation (HCD) at a normalized collision energy of 29%, and fragment ions were analyzed in the linear ion trap. Dynamic exclusion was set to 30 s after two MS^2^ events.

MS/MS data were processed using Mascot (version 3.0, Matrix Science, London, UK) and searched against the UniProt *Bos taurus* database. Search parameters included a precursor mass tolerance of 10 ppm and fragment mass tolerance of 0.60 Da. Peptide identifications were validated using Scaffold (version 5.3.3; Proteome Software Inc., Portland, OR, USA) and accepted at a peptide probability > 90% with an estimated false discovery rate (FDR) ≤ 1%.

Bioactivity prediction of identified peptide sequences was performed using UniDL4BioPep (Du et al. 2023), available at https://nepc2pvmzy.us‐east‐1.awsapprunner.com/ (accessed June 15, 2025). This is a deep learning‐based framework for peptide bioactivity classification. Peptide sequences in single‐letter amino acid notation were converted into numerical embeddings derived from pre‐trained protein language models and classified using a convolutional neural network architecture. Peptides with predicted probability ≥ 0.5 were considered potentially bioactive. Identified sequences were further screened against public databases to assess novelty and identify previously unreported peptides.

### 2.11 Statistical Analysis

All experiments were performed in triplicate, and the results were processed by analysis of variance (ANOVA), followed by Tukey's multiple comparison test at a significance level of *p* < 0.05. Statistical analyses were conducted using GraphPad Prism 8.0 (GraphPad Software, San Diego, CA, USA).

## 3 Results and Discussion

### 3.1 Proteolytic Activity in Requeson Cheese

**FIGURE 1:**
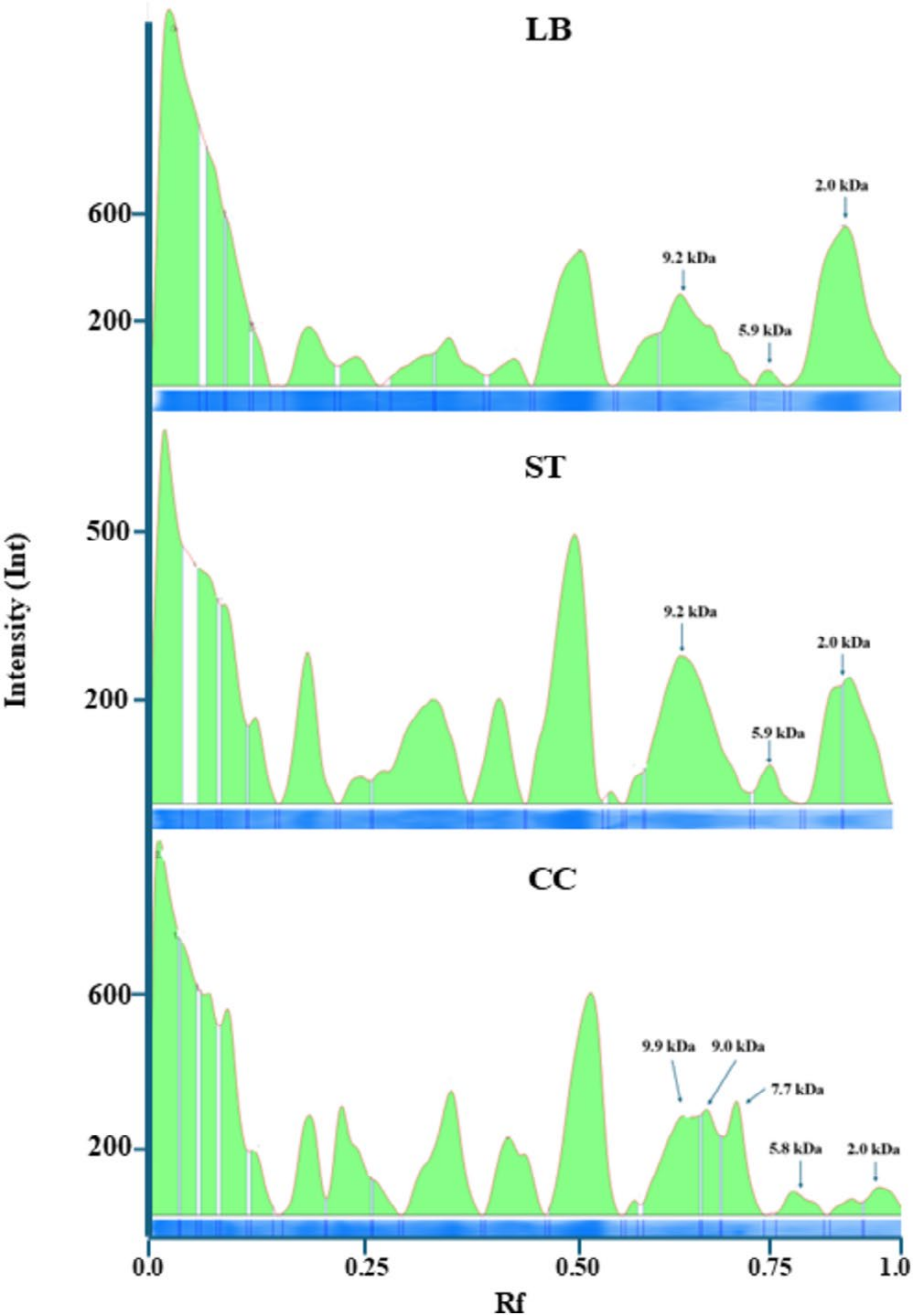
SDS‐PAGE peptide profiles of Requeson cheese produced from whey fermented with *L. bulgaricus* (LB), *S. thermophilus* (ST), and their co‐culture (CC).

The proteolytic capacity of lactic acid bacteria has been widely explored due to its relevance in food processing and its potential for generating bioactive compounds with therapeutic properties, such as peptides (Akbal et al. 2025). Moreover, although both strains used in this study ( *L. bulgaricus* 2772 and *S. thermophilus* SY‐102) have been previously reported to produce bioactive peptides (Sebastián‐Nicolas et al. 2021; Guzmán‐Rodríguez et al. 2024), their proteolytic activity has so far been evaluated only in culture media and not in a structured dairy product such as Requeson cheese. In the present study, the proteolytic performance of each fermentation system was determined by measuring the concentration of free amino groups (Table 1) and by analyzing peptide profiles in the final product using electrophoretic techniques (Figure 1).

**TABLE 1:** Quantification of free amino groups in Requeson cheese formulations.

Formulation	Free amino group (μg eq of glycine/g of Requeson cheese)
LB Requeson	249 ± 8.07^b^
ST Requeson	226 ± 15.52^c^
CC Requeson	295 ± 10.21^a^

*Note:* The values shown represent averages of triplicate samples (data are mean ± SD). Different lowercase letters indicate a significant difference in the same column (*p* < 0.05).

Abbreviations: CC, Co‐culture; LB, *L. delbrueckii* ssp *bulgaricus*; ST, *S. thermophilus* .

Monoculture systems of *L. bulgaricus* or *S. thermophilus* showed comparable concentrations of free amino groups, which is consistent with previous findings under similar nutrient and temperature conditions (Chelladhurai et al. 2025). Nevertheless, *L. bulgaricus* exhibited slightly higher proteolytic activity, as expected, due to its greater capacity to hydrolyze whey proteins (Song et al. 2021). In contrast, the co‐culture system exhibited a significantly greater release of amino groups, likely due to the synergistic interaction between both strains, which has been previously reported to enhance proteolysis (Wu et al. 2025).

Peptide profiling by Tris‐Tricine‐SDS‐PAGE (Figure 1) revealed the presence of low‐molecular‐weight peptides (< 10 kDa) across all fermented samples, which have been associated with diverse biological activities (Helal, Pierri, et al. 2023). The monoculture systems presented similar electrophoretic patterns, while the co‐culture displayed a distinct profile, likely influenced by the increased degree of hydrolysis. This divergence in peptide composition may contribute to differences in the nature and intensity of the observed bioactivities (Jiang et al. 2025).

### 3.2 Antioxidant Activity

**FIGURE 2:**
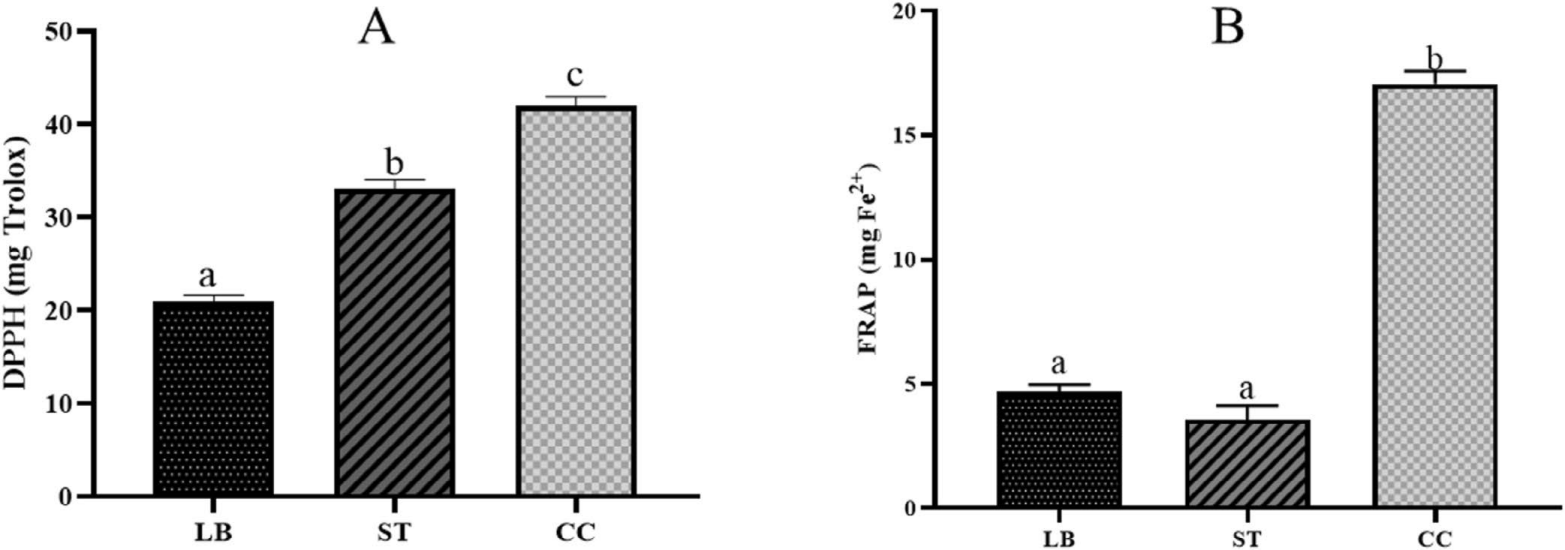
Antioxidant activity of Requeson cheese formulations evaluated by DPPH (A) and FRAP (B), with *L. bulgaricus* (LB), *S. thermophilus* (ST), and co‐culture (CC). Different letters indicate statistically significant differences (*p* < 0.05).

There is growing interest in investigating the antioxidant properties of several compounds, as these can aid in managing various pathophysiological conditions in the human body by preventing the propagation of free radicals and reactive oxygen species (de Espindola et al. 2023). The results of the antioxidant assays are shown in Figure 2.

Both assays detected measurable effects in all samples; however, the highest values were observed in the co‐culture system compared to the monocultures. The discrepancies between the two assays may be attributed to differences in peptide polarity, as polar residues favor electron transfer mechanisms. Consequently, the lower FRAP values may reflect a lack of polar residues in the peptide structures (Alfaia et al. 2024). In contrast, the higher radical‐scavenging activity observed in the DPPH assay may indicate the presence of aromatic amino acids, which are known to enhance this antioxidant mechanism (Perlikowska et al. 2022).

The results are consistent with those of Heydari et al. (2023), who found that a co‐culture of *Lactobacillus delbrueckii* subsp. *bulgaricus*, *S. thermophilus* , and *Bifidobacterium lactis* had the highest antioxidant activity in a fermented yoghurt‐type food. Comparable studies have reported the antioxidant activity in peptides obtained by fermentation with different microorganisms, such as *Limosilactobacillus reuteri* WQ‐Y1 (Cui et al. 2022), a co‐culture of *L. fermentum* and *S. cerevisiae* (Dineshbhai et al. 2022), *Lacticaseibacillus rhamnosus* NCDC24 (Srivastava et al. 2022), *Lactobacillus rhamnosus* B2‐1 (Guo et al. 2024), and *S. thermophilus* RBC06 (Solieri et al. 2022).

Regarding whey cheeses specifically, Akan et al. (2021) concluded that the antioxidant activity, as evaluated by the DPPH method, in a Turkish whey cheese (Lor) could be attributed to peptides derived from proteolysis carried out by the post‐manufacture microbiota. Similar antioxidant levels were maintained even after 20 days of storage. Peptides with antioxidant activity often contain hydrophobic or aromatic residues. Additionally, their antioxidant capacity may be enhanced when the structure contains these amino acid repeats in the peptide chain. Although this structure is frequently observed in β‐lactoglobulin (the primary source of antioxidant whey protein peptides), several α‐lactalbumin derivatives exhibiting similar properties have been previously reported (Corrochano, Buckin, et al. 2018).

Finally, it has been reported that heat treatment can enhance the antioxidant capacity of whey proteins by inducing structural changes that expose thiol groups, which then react with reactive oxygen species (Arranz et al. 2019). Therefore, the thermal treatment applied during Requeson cheese production (95°C for 1 h) may have also contributed to the antioxidant activity observed in this study.

### 3.3 Mineral Chelating Activity

**FIGURE 3:**
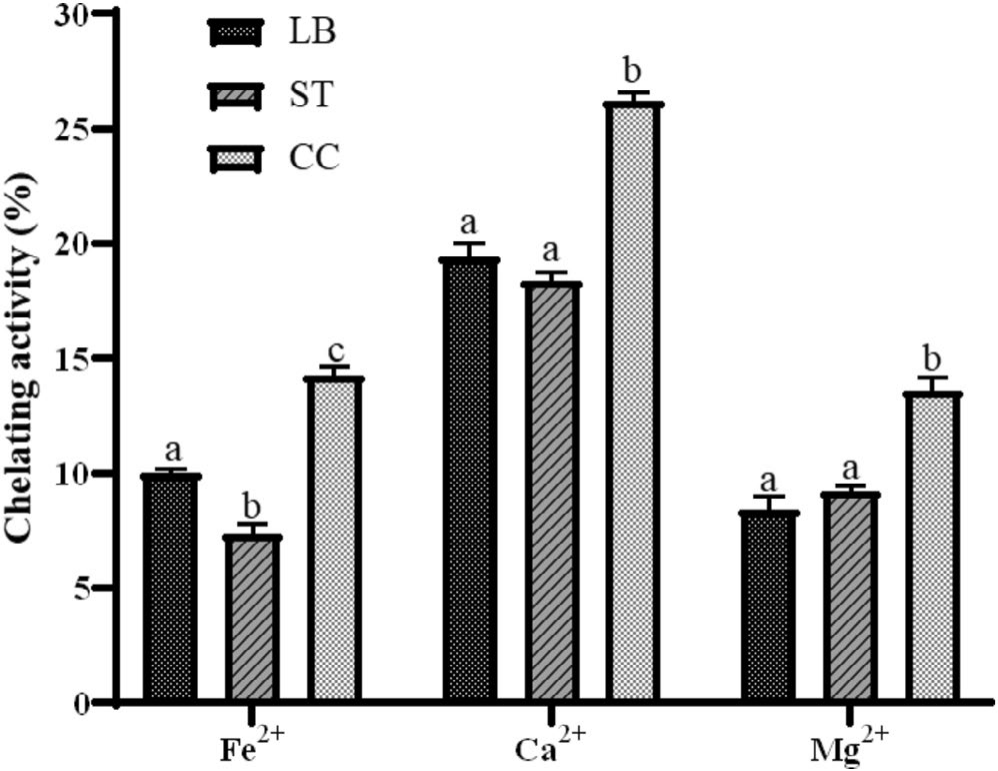
Mineral chelating activity of Requeson cheese formulations for iron (Fe^2+^), calcium (Ca^2+^), and magnesium (Mg^2+^), with *L. bulgaricus* (LB), *S. thermophilus* (ST), and co‐culture (CC). Different letters indicate statistically significant differences (*p* < 0.05).

The focus on studying mineral chelating peptides stems mainly from a public health strategy derived from interest in foods with molecules capable of enhancing the transport of minerals in the gastrointestinal tract and improving their absorption in the body, which could be paired with the treatment of anemia or osteoporosis for an improved outcome (Canabady‐Rochelle et al. 2018). The chelating capacities for iron, calcium, and magnesium are shown in Figure 3.

Among the minerals studied, calcium showed the highest chelating capacity, followed by iron, while magnesium exhibited the lowest. Co‐culture samples recorded the highest chelation levels, aligning with findings by Liu et al. (2024), who reported enhanced Ca^2+^ chelation in peptides produced by a co‐culture of *Lactiplantibacillus plantarum* and *Lacticaseibacillus casei* (1:1) compared to monocultures. This increase is attributed not only to peptide presence but also to their chemical structures, which allow diverse interactions with divalent ions via coordination bonds.

Differences in iron and calcium chelation have also been reported by González‐Olivares et al. (2023), linking these effects to peptides from cow milk caseins, especially β‐casein, which can form during sweet whey pretreatment (Corrochano, Arranz, et al. 2018). Similarly, Athira et al. (2021) observed that whey peptides hydrolyzed with alcalase exhibited a chelating capacity of 36.42 μg Fe^2+^/mg protein, attributed to residues like tyrosine, tryptophan, methionine, lysine, and cysteine. These residues also contribute to antioxidant activity via electron transport mechanisms.

Accordingly, Fe^2+^‐chelated peptides may enhance FRAP‐measured antioxidant activity. González‐Olivares et al. (2023) observed a 400% increase in antioxidant capacity for Fe^2+^ chelated whey peptides compared to non‐chelated controls. Wu et al. (2024) similarly reported up to 85.92% chelating capacity in < 3 kDa peptide fractions from trypsin‐treated whey proteins, emphasizing the importance of peptide‐to‐salt ratios, reaction time, and temperature in optimizing chelation.

Amino acid composition is also critical in iron chelation, particularly in peptides from β‐LG (Caetano‐Silva et al. 2020). Residues such as Asp, Glu, His, and Leu form stable coordination compounds through carboxyl, amino, and imidazole groups, similar to peptides from mung bean (Zhang et al. 2021). Calcium chelation shows comparable patterns, with Tyr and Asp playing key roles in complex formation via terminal amino and carboxyl groups (Wang et al. 2018).

Additional studies (Hou et al. 2018; An et al. 2022) highlighted that phosphate groups enhance calcium binding due to their negative charge and multidentate coordination, with the number of phosphate groups affecting the complex's stability and coordination mode (mono‐, bi‐, or tridentate).

Although 80%–90% of magnesium is lost during food processing, studies on magnesium‐chelating peptides remain limited (Szymoniak et al. 2022). Cao et al. (2017) reported that casein phosphopeptides—originally purified for Ca^2+^ affinity—also chelated Mg^2+^ at 78.4 μg/300 μg peptide in Caco‐2 cells. Similarly, Zhang, Du, et al. (2023) found that peptides from bovine bone proteins treated with alcalase and papain formed stable Mg^2+^ complexes, achieving 21.56 ± 0.42 mg Mg^2+^/g protein. Chelation was attributed to glycine flexibility, electrostatic interactions involving Glu, Asp, Arg, Lys, His, and π‐type interactions between Phe or Tyr and magnesium ions.

### 3.4 Angiotensin Converting Enzyme (ACE‐I) Inhibitory Activity

**FIGURE 4:**
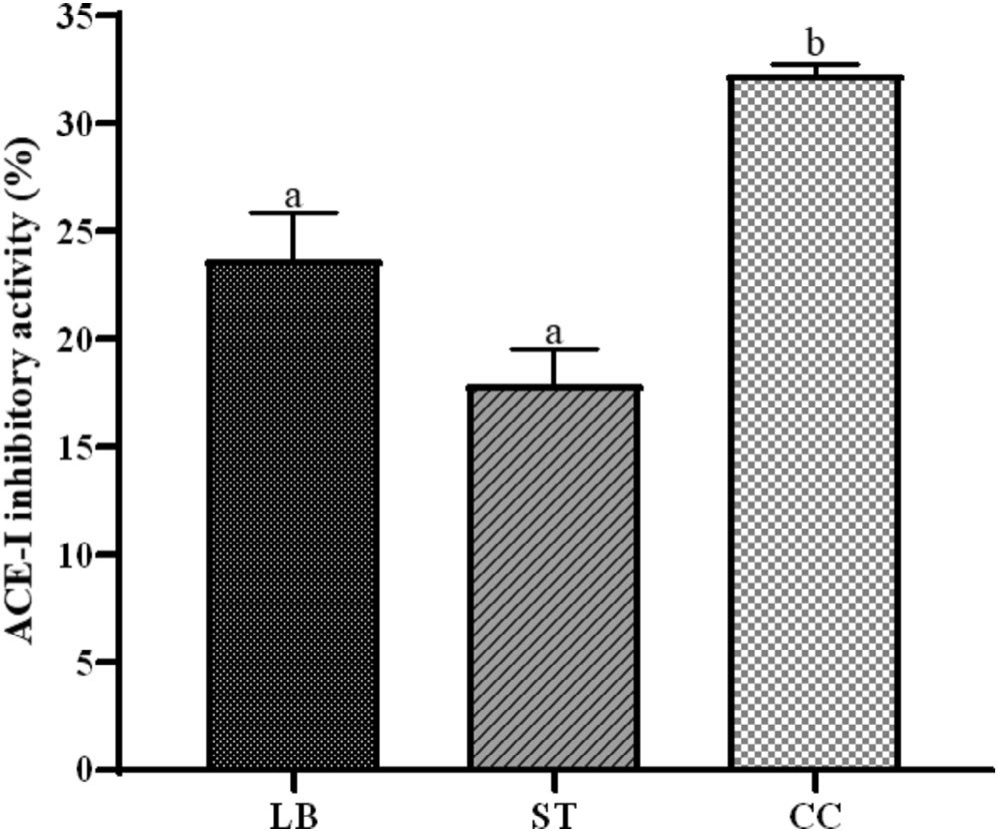
Angiotensin‐converting enzyme (ACE‐I) inhibitory activity (%) of Requeson cheese formulations: *L. bulgaricus* (LB), *S. thermophilus* (ST), and co‐culture (CC). Different letters indicate statistically significant differences (*p* < 0.05).

Conditions associated with high blood pressure are having an increasing impact on annual human mortality (Jimenez et al. 2024). Therefore, the identification of peptides capable of complementing existing pharmacological and dietary interventions is of significant interest. The antihypertensive capacity of each sample, expressed as percentage inhibition of angiotensin I‐converting enzyme (ACE‐I), is presented in Figure 4.

ACE inhibition was observed in all samples examined, with those from the co‐culture exhibiting the highest activity. Several quantitative structure–activity relationship (QSAR) studies have attributed this activity to peptides containing hydrophobic amino acids at their C‐terminal end, such as proline, tyrosine, leucine, and tryptophan (Yao et al. 2024). This feature is generally found in peptides with antihypertensive activity by ACE inhibition and is therefore related to peptides generated during whey fermentation in each of the systems assessed. Ramírez‐Rivas et al. (2022) studied ACE‐I inhibition in Requeson cheese and found that the increasing addition of NaCl diminished the activity of interest while also attributing the presence of this activity to the proteolysis they found in the artisanal sweet whey derived from their native microbiota, since their whey was collected directly from a cheese factory without pasteurization. In another study, Daliri et al. (2018) found that *Pediococcus acidilactici* SDL1414 produced peptides from whey proteins with an ACE‐I inhibition of 84.70%. These peptides had molecular weights under 7 kDa, and their structures contained homologous sequences to those of previously studied antihypertensive peptides. Consistent with this, Mazorra‐Manzano et al. (2020) found that by incubating sweet whey with its native microbiota, ACE‐I inhibitory activity increased from 20% in unfermented whey to 70% inhibition after incubation for 120 h at 37°C.

Comparisons with related studies from our group support this pattern. Sebastián‐Nicolas et al. (2021) reported 80.6% ACE‐I inhibition after 21 h of fermentation using *S. thermophilus* SY‐102 co‐cultured with *L. rhamnosus* GG. Similarly, Olvera‐Rosales, Pérez‐Escalante, et al. (2023) observed 52.42% inhibition with the same strains. Guzmán‐Rodríguez et al. (2024) found that fermenting milk with *L. bulgaricus* NCFB 2772 and *L. rhamnosus* GG for 12 h yielded 77.6% ACE‐I inhibition. These findings highlight the influence of strain compatibility and nutrient requirements on the generation and efficacy of bioactive peptides, particularly those with antihypertensive potential. Peptides such as Val‐Pro‐Pro and Ile‐Pro‐Pro‐Pro have been consistently associated with effective ACE‐I inhibition (Hussein et al. 2020).

### 3.5 Inhibition of the Dipeptidyl Peptidase IV (DPP‐IV) Enzyme

**FIGURE 5:**
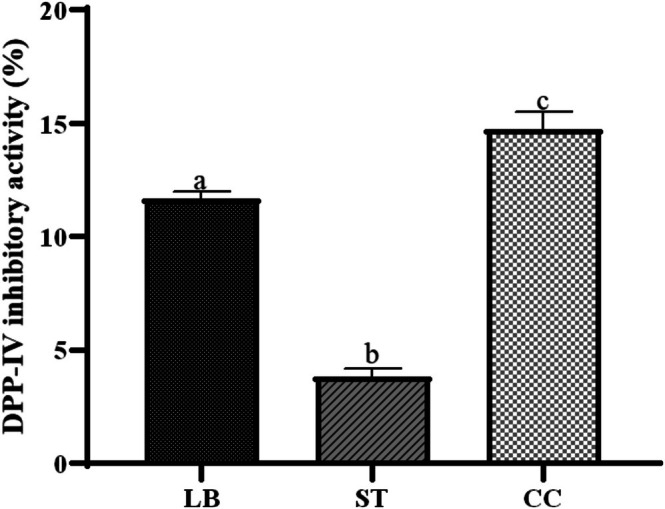
DPP‐IV enzyme inhibition (%) of Requeson cheese formulations: *L. bulgaricus* (LB), *S. thermophilus* (ST), and co‐culture (CC). Different letters indicate statistically significant differences (*p* < 0.05).

One of the most recent approaches for managing Type 2 *Diabetes mellitus* involves targeting the dipeptidyl peptidase IV (DPP‐IV) enzyme, which regulates the incretin hormones, such as GLP‐1 and GIP. These hormones stimulate postprandial insulin secretion, leading to the use of DPP‐IV inhibitors to improve the patient's glycemic control. However, some drugs that exert this function have been associated with undesirable side effects (Zaresharifi et al. 2024). For this reason, bioactive peptides with the ability to inhibit DPP‐IV have been investigated as a complement to these drugs (Sato et al. 2018). Whey proteins have proven to be an outstanding source for these types of compounds (Jia et al. 2020), generating interest in further exploring different mechanisms to obtain them from this source (Elisha et al. 2025). The DPP‐IV inhibitory activity of the samples studied is presented in Figure 5.

The most notable finding was the significantly higher DPP‐IV inhibition in co‐culture samples compared to monocultures, consistent with other bioactivities observed. However, microbial protocooperation does not always yield peptides with superior activity. Olvera‐Rosales, Cruz‐Guerrero, et al. (2023) reported lower DPP‐IV inhibition in a co‐culture of *L. rhamnosus* GG and *S. thermophilus* SY‐102 (55.49%) compared to *L. rhamnosus* GG alone (63.3%). This was attributed to peptides produced by *S. thermophilus* during whey protein hydrolysis. The microorganism type and its proteolytic system are key determinants of peptide bioactivity (Ge et al. 2024).

Other studies have also demonstrated DPP‐IV inhibition by whey‐derived peptides; for example, *Enterococcus faecalis* 2/28 fermentation yielded 43.8% inhibition (Worsztynowicz et al. 2020). QSAR analyses indicate that hydrophobic residues in the N‐terminal or penultimate position enhance DPP‐IV inhibition by interacting with the enzyme's active site, particularly Ser630, via hydrophobic and hydrogen bonds (Hrynkiewicz et al. 2023). Accordingly, Pro, Ala, Val, Leu, and Phe are frequently found in DPP‐IV inhibitory peptides (Zhang, Zhu, et al. 2023).

### 3.6 Antimicrobial Activity

**FIGURE 6:**
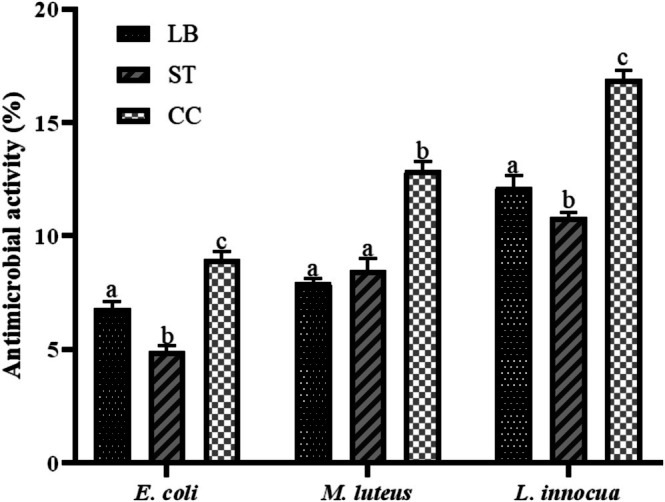
Antimicrobial activity (%) of Requeson cheese formulations against *Escherichia coli* (K12), *Micrococcus luteus* (CD‐BBB1018), and *Listeria innocua* (ATCC) 33,090. Treatments: Fermentation with *L. bulgaricus* (LB), *S. thermophilus* (ST), and co‐culture (CC). Different letters indicate statistically significant differences (*p* < 0.05) between formulations within the same bacterial strain.

Lactic acid bacteria are increasingly recognized as sources of antimicrobial compounds, with potential applications as natural food preservatives and complementary strategies against antimicrobial resistance (Ghailan and Niamah 2025). Among these bioactive compounds, peptides derived from milk proteins have been reported to display antimicrobial activity against both bacterial and fungal pathogens (Ashokbhai et al. 2022), prompting their screening in the Requeson cheese formulations developed. The percentage inhibition observed for each indicator microorganism is shown in Figure 6.

The highest levels of inhibition were observed against *L. innocua* and *M. luteus* , while *E. coli* showed lower susceptibility. This agrees with the report by Innocente et al. (2023), who found 31.07% inhibition against *E. coli* and 84.13% against *L. monocytogenes* in whey protein hydrolysates generated from an alcalase treatment for 120 min. The lower inhibition of *E. coli* , a Gram‐negative bacterium, may be due to its protective outer membrane and periplasmic space, which reduce the efficacy of peptide‐based disruption (Hetta et al. 2024).

While many antimicrobial peptides (AMPs) have been identified as derivatives of α‐lactalbumin and β‐lactoglobulin (Guha et al. 2021), others that originate from alternative whey proteins have also been reported. For instance, peptides resulting from the hydrolysis of camel milk lactoferrin have demonstrated antimicrobial activity. Alzain et al. (2024) described peptide P33, which was active against methicillin‐resistant *Staphylococcus aureus* , with a minimum inhibitory concentration of 2.083 mg/mL. This activity was attributed to the peptide's ability to inhibit the SauPBP2a protein, which plays a key role in peptidoglycan synthesis within the bacterial cell wall.

Similar studies have described peptides derived from trypsin‐treated buffalo milk whey and found that two of them, derived from β‐lactoglobulin, showed bacteriostatic activity against *E. coli* (82.7%), *S. aureus* (81.8%), *S. typhimurium* (81.5%), and *L. monocytogenes* (80.5%) with a concentration of 512 μg/mL. These activities have been related to electrostatic interactions between the residues at the N‐ and C‐terminus of the peptide and the lipopolysaccharide of the cell structure of the microorganism (Talapko et al. 2022). Importantly, AMPs typically require short peptide chains to permeate bacterial membranes and disrupt physiological processes essential to microbial survival (Kapil and Sharma 2021).

Overall, these results support that whey‐derived peptides generated via microbial co‐culture have considerable potential as multifunctional bioactive agents. Beyond their antimicrobial properties, they may also contribute antioxidant, antihypertensive, antidiabetic, and immunomodulatory effects, positioning them as promising functional ingredients for novel therapeutic and nutritional applications.

While the present findings demonstrate in vitro growth inhibition, additional studies, including determination of minimum inhibitory concentration (MIC), stability during storage, and resistance to gastrointestinal digestion, are necessary to fully assess the functional applicability of these peptides in the final product.

### 3.7 Peptide Screening and Functional Prediction

Following the evaluation of biological activities in the Requeson formulations, peptides were identified via LC–MS/MS. The sequences were analyzed for predicted bioactivities using the UniDL4BioPep web server, which employs a pre‐trained language model and a CNN trained on peptide datasets (Du et al. 2023). Notably, the current model does not predict mineral‐chelating activity; however, some studies suggest antioxidant peptides may also chelate minerals due to shared mechanisms (Zhang, Du, et al. 2023). Peptides with a predicted activity probability ≥ 0.5 are listed in Table 2. Eleven peptides were predicted to exhibit antioxidant activity, 11 showed ACE‐I inhibition, 6 had potential DPP‐IV inhibition, and 3 displayed antimicrobial properties.

**TABLE 2:** Predicted bioactive peptides identified in Requeson cheese formulations fermented with *L. bulgaricus* , *S. thermophilus* , and their co‐culture.

Precursor protein	Sequence	Predicted activity^a^	Presence
*α‐LA*	ALCSEKLDQWLCEKL	Antioxidant	LB, ST, CC
	FLDDDLTDDIMCVK	Antioxidant	LB, ST, CC
	CEVFRELKDLK	DPP‐IV inhibitor	ST, CC
*β‐LG*	ALKALPMHIR	ACE‐I inhibitor Antimicrobial	LB, CC
	IPAVFKIDALNENK	ACE‐I inhibitor	LB, CC
	LSFNPTQLEEQCHI	ACE‐I inhibitor	LB, ST, CC
	TKIPAVFK	ACE‐I inhibitor	LB, ST, CC
*LF*	CSTSPLLEACAFLTR	Antioxidant	ST, CC
	AFALECIR	DPP‐IV inhibitor	CC
	YYGYTGAFR	Antioxidant	ST, CC
	LGAPSITCVR	Antimicrobial	LB, ST, CC
	GSNFQLDQLQGR	DPP‐IV inhibitor	LB, CC
*κ‐CN*	FFSDKIAK	Antimicrobial	LB, ST, CC
	SCQAQPTTMAR	Antioxidant	LB, ST, CC
*PP3*	SSRQPQSQNPK	ACE‐I inhibitor	LB, ST, CC
	LPLSILKEK	DPP‐IV inhibitor	LB, ST, CC
*LP*	VGPLLACLLGR	Antioxidant	LB, ST, CC
	SPALGAANR	DPP‐IV inhibitor	CC
	GFCGLSQPK	ACE‐I inhibitor	CC
*β‐CN*	DMPIQAFLL	DPP‐IV inhibitor	LB, ST, CC
	HKEMPFPK	Antioxidant	ST, CC
	DMPIQAFLLYQEPVLGPVR	ACE‐I inhibitor	LB, ST, CC
	VKEAMAPK	ACE‐I inhibitor	LB, ST, CC
	VLPVPQK	ACE‐I inhibitor	ST, CC
*αs1‐CN*	HQGLPQEVLNENLLR	Antioxidant	LB, ST, CC
	FFVAPFPEVFGKEK	ACE‐I inhibitor	LB, ST, CC
	LHSMKEGIHAQQK	Antioxidant	LB, ST, CC
	EPMIGVNQELAYFYPELFR	Antioxidant	ST, CC
	YLGYLEQLLRLK	Antioxidant	LB, ST, CC
*αS2‐CN*	TVYQHQK	ACE‐I inhibitor	ST, CC

Abbreviations: α‐LA, α‐Lactalbumin; αs1‐CN, αs1‐cassein; αs2‐CN, αs2‐casein; β‐CN, β‐casein; β‐LG, β‐Lactoglobulin; κ‐CN, kappa casein; CC, Co‐culture; LB, *L. delbrueckii* ssp. *bulgaricus*; LF, Lactoferrin; LP, Lactoperoxidase; PP3, Proteose‐peptone component 3; ST, *S. thermophilus* .

^a^ Biological activity predicted using the UniDL4BioPep server (Du et al. 2023).

All peptides identified through LC–MS/MS were present in the co‐culture formulation, whereas only a subset of these sequences appeared in the monoculture samples. This discrepancy is likely related to the distinct proteolytic systems present in the bacterial strains used, as discussed in Section 3.1. Each bacterium possesses unique metabolic and nutritional requirements that affect protease expression, cleavage site specificity, and consequently, the peptides generated (Rodríguez‐Serrano et al. 2018).

The predominance of antioxidant and ACE‐I inhibitory peptides in the co‐culture system has also been reported in other systems fermented by *Lactobacillus* species, likely due to their ability to cleave proteins at specific sites, generating peptides enriched in hydrophobic amino acids (Helal, Nasuti, et al. 2023). Furthermore, other studies (Cui et al. 2022) have noted that peptides produced by LAB containing residues such as proline, valine, and serine tend to exhibit enhanced radical‐scavenging activity. These amino acids were found in most of the sequences identified as having antioxidant activity in our samples.

The observed ACE‐I inhibitory activity may be attributed to the broad spectrum of enzymatic activities present in the different fermentation systems (AlKhalidy and Dosh 2023). In *S. thermophilus* , the activities of extracellular, cell wall‐associated enzymes such as aminopeptidases, carboxypeptidases, peptidyl‐dipeptidases, and X‐prolyl dipeptidyl‐peptidases have been linked to the hydrolysis of proline‐rich encrypted sequences, which are commonly associated with this bioactivity (Solieri et al. 2022).

DPP‐IV inhibitory peptides are characterized by the presence of basic and hydrophobic amino acids; however, an excessive presence of basic residues may reduce binding affinity to the enzyme's active site, thereby diminishing inhibitory potential (Zhou et al. 2024). Basic amino acids are also essential structural components of antimicrobial peptides, facilitating electrostatic interactions with the negatively charged surfaces of bacterial membranes. This interaction disrupts membrane integrity, leading to permeabilization and subsequent cell death (Álvarez et al. 2024).

Finally, chemical synthesis of the identified peptide sequences would enable experimental validation of the predicted bioactivities and further elucidation of the mechanisms underlying the in vitro effects observed in Requeson cheese extracts (Guzmán et al. 2021).

## 4 Conclusions

Requeson cheese produced through the fermentation of sweet whey with two lactic acid bacteria strains demonstrated enhanced in vitro functional properties compared to non‐fermented controls. Among the fermentation strategies evaluated, the co‐culture of *Lactobacillus delbrueckii* subsp. *bulgaricus* and *Streptococcus thermophilus* exhibited significantly higher proteolytic activity and bioactivity in the assays performed relative to monoculture systems.

It should be emphasized that the biological activities reported herein were assessed exclusively through in vitro assays. Therefore, further research is required to confirm the efficacy, bioavailability, and safety of the identified peptide candidates through chemical characterization and in vivo studies. Additionally, evaluation of peptide stability during storage, sensory acceptability, and compliance with regulatory frameworks governing functional food claims will be necessary prior to industrial application.

Overall, the present findings support the valorization of sweet whey as a substrate for the development of fermented dairy products with enhanced functional potential, contributing to more sustainable utilization of dairy by‐products.

## Author Contributions

**Luis‐Fernando Patlan‐Velázquez:** data curation, investigation, writing – original draft, conceptualization. **Ulises Carrasco‐Navarro:** software, methodology. **Lorena Gómez‐Ruiz:** validation, investigation. **Mariano García‐Garibay:** funding acquisition. **Sergio Alatorre‐Santamaría:** formal analysis, methodology. **Gabriela Rodríguez‐Serrano:** formal analysis, project administration. **Alma Cruz‐Guerrero:** project administration, methodology, supervision. **Luis‐Guillermo González‐Olivares:** writing – review and editing.

## Conflicts of Interest

The authors declare no conflicts of interest.

## Data Availability

The data that support the findings of this study are available from the corresponding author upon reasonable request.

## References

[fsn371806-bib-0001] Akan, E. , O. Yerlikaya , A. Akpinar , C. Karagozlu , O. Kinik , and H. R. Uysal . 2021. “The Effect of Various Herbs and Packaging Material on Antioxidant Activity and Colour Parameters of Whey (LOR) Cheese.” International Journal of Dairy Technology 74, no. 3: 554–563. 10.1111/1471-0307.12778.

[fsn371806-bib-0002] Akbal, S. , E. Uğur Geçer , and P. Ertürkmen . 2025. “Probiotic Viability and Bioactive Properties of Buffalo Yoghurt Produced Using High Cholesterol‐Assimilating Probiotic Strains.” Veterinary Medicine and Science 11, no. 2: e70233. 10.1002/vms3.70233. 39912884 PMC11800370

[fsn371806-bib-0003] Akbarian, M. , A. Khani , S. Eghbalpour , and V. N. Uversky . 2022. “Bioactive Peptides: Synthesis, Sources, Applications, and Proposed Mechanisms of Action.” International Journal of Molecular Sciences 23, no. 3: 1445. 10.3390/ijms23031445. 35163367 PMC8836030

[fsn371806-bib-0004] Alfaia, C. M. , L. Patarata , and M. J. d. R. Fraqueza . 2024. “Antioxidant Compounds From Fermentation and Microbial Sources.” In Natural Antioxidants to Enhance the Shelf‐Life of Food, edited by M. Pateiro , 215–252. Academic Press.

[fsn371806-bib-0005] AlKhalidy, S. J. , and K. S. Dosh . 2023. “Isolation and Purification of αs‐CN From Sheep Milk and Measuring the Effectiveness of Its Enzymatic Hydrolysis in Inhibiting ACE1.” Chemical Methodologies 7, no. 2: 156–166. 10.22034/chemm.2023.366342.1618.

[fsn371806-bib-0006] Álvarez, C. A. , T. Toro‐Araneda , J. P. Cumillaf , et al. 2024. “Evaluation of the Biological Activities of Peptides From Epidermal Mucus of Marine Fish Species From Chilean Aquaculture.” Marine Drugs 22, no. 6: 248. 10.3390/md22060248. 38921559 PMC11204461

[fsn371806-bib-0007] Alzain, M. , E. M. M. Ali , M. Zamzami , et al. 2024. “Identification of Antimicrobial Bioactive Peptides From the Camel Milk Protein Lactoferrin: Molecular Docking, Molecular Dynamic Simulation, and In Vitro Study.” Food and Humanity 3: 100414. 10.1016/j.foohum.2024.100414.

[fsn371806-bib-0008] An, J. , Y. Zhang , Z. Ying , et al. 2022. “The Formation, Structural Characteristics, Absorption Pathways and Bioavailability of Calcium‐Peptide Chelates.” Food 11, no. 18: 2762. 10.3390/foods11182762. PMC9497609 36140890

[fsn371806-bib-0009] Arranz, E. , A. R. Corrochano , C. Shanahan , et al. 2019. “Antioxidant Activity and Characterization of Whey Protein‐Based Beverages: Effect of Shelf Life and Gastrointestinal Transit on Bioactivity.” Innovative Food Science & Emerging Technologies: IFSET: The Official Scientific Journal of the European Federation of Food Science and Technology 57: 102209. 10.1016/j.ifset.2019.102209.

[fsn371806-bib-0010] Ashokbhai, J. K. , B. Basaiawmoit , S. Das , et al. 2022. “Antioxidative, Antimicrobial and Anti‐Inflammatory Activities and Release of Ultra‐Filtered Antioxidative and Antimicrobial Peptides During Fermentation of Sheep Milk: In‐Vitro, In‐Silico and Molecular Interaction Studies.” Food Bioscience 47: 101666. 10.1016/j.fbio.2022.101666.

[fsn371806-bib-0011] Athira, S. , B. Mann , R. Sharma , R. Pothuraju , and R. K. Bajaj . 2021. “Preparation and Characterization of Iron‐Chelating Peptides From Whey Protein: An Alternative Approach for Chemical Iron Fortification.” Food Research International 141: 110133. 10.1016/j.foodres.2021.110133. 33642000

[fsn371806-bib-0012] Bintsis, T. , and P. Papademas . 2023. “Sustainable Approaches in Whey Cheese Production: A Review.” Dairy 4, no. 2: 249–270. 10.3390/dairy4020018.

[fsn371806-bib-0013] Bradford, M. M. 1976. “A Rapid and Sensitive Method for the Quantitation of Microgram Quantities of Protein Utilizing the Principle of Protein‐Dye Binding.” Analytical Biochemistry 72: 248–254. 10.1006/abio.1976.9999. 942051

[fsn371806-bib-0014] Caetano‐Silva, M. E. , F. M. Simabuco , R. M. N. Bezerra , et al. 2020. “Isolation and Sequencing of Cu‐, Fe‐, and Zn‐ Binding Whey Peptides for Potential Neuroprotective Applications as Multitargeted Compounds.” Journal of Agricultural and Food Chemistry 68, no. 44: 12433–12443. 10.1021/acs.jafc.0c03647. 33095576

[fsn371806-bib-0015] Canabady‐Rochelle, L. L. S. , K. Selmeczi , S. Collin , A. Pasc , L. Muhr , and S. Boschi‐Muller . 2018. “SPR Screening of Metal Chelating Peptides in a Hydrolysate for Their Antioxidant Properties.” Food Chemistry 239: 478–485. 10.1016/j.foodchem.2017.06.116. 28873593

[fsn371806-bib-0016] Candiano, G. , M. Bruschi , L. Musante , et al. 2004. “Blue Silver: A Very Sensitive Colloidal Coomassie G‐250 Staining for Proteome Analysis.” Electrophoresis 25, no. 9: 1327–1333. 10.1002/elps.200305844. 15174055

[fsn371806-bib-0017] Cao, Y. , J. Miao , G. Liu , et al. 2017. “Bioactive Peptides Isolated From Casein Phosphopeptides Enhance Calcium and Magnesium Uptake in Caco‐2 Cell Monolayers.” Journal of Agricultural and Food Chemistry 65, no. 11: 2307–2314. 10.1021/acs.jafc.6b05711. 28218527

[fsn371806-bib-0018] Chai, K. F. , A. Y. H. Voo , and W. N. Chen . 2020. “Bioactive Peptides From Food Fermentation: A Comprehensive Review of Their Sources, Bioactivities, Applications, and Future Development.” Comprehensive Reviews in Food Science and Food Safety 19, no. 6: 3825–3885. 10.1111/1541-4337.12651. 33337042

[fsn371806-bib-0019] Chelladhurai, K. , S. Warakaulle , S. N. Ali , M. S. Turner , M. Ayyash , and A. Kamal‐Eldin . 2025. “Differences in the Growth, Acidification, and Proteolytic Activities of *Lactobacillus helveticus* , *Lactobacillus delbrueckii* Subsp. *Bulgaricus*, and *Streptococcus thermophilus* in Camel and Cow Milk Fermentation.” International Dairy Journal 160: 106075. 10.1016/j.idairyj.2024.106075.

[fsn371806-bib-0020] Corrochano, A. R. , E. Arranz , I. De Noni , et al. 2018. “Intestinal Health Benefits of Bovine Whey Proteins After Simulated Gastrointestinal Digestion.” Journal of Functional Foods 49: 526–535. 10.1016/j.jff.2018.08.043.

[fsn371806-bib-0021] Corrochano, A. R. , V. Buckin , P. M. Kelly , and L. Giblin . 2018. “Invited Review: Whey Proteins as Antioxidants and Promoters of Cellular Antioxidant Pathways.” Journal of Dairy Science 101, no. 6: 4747–4761. 10.3168/jds.2017-13618. 29605324

[fsn371806-bib-0022] Cui, L. , G. Yang , S. Lu , et al. 2022. “Antioxidant Peptides Derived From Hydrolyzed Milk Proteins by *Lactobacillus* Strains: A BIOPEP‐UWM Database‐Based Analysis.” Food Research International 156, no. 111339: 111339. 10.1016/j.foodres.2022.111339. 35651087

[fsn371806-bib-0023] Daliri, E. , B. Lee , B. Park , S. Kim , and D. Oh . 2018. “Antihypertensive Peptides From Whey Proteins Fermented by Lactic Acid Bacteria.” Food Science and Biotechnology 27, no. 6: 1781–1789. 10.1007/s10068-018-0423-0. 30483443 PMC6233406

[fsn371806-bib-0024] de Espindola, J. S. , M. Ferreira Taccóla , V. S. da Silva , et al. 2023. “Digestion‐Resistant Whey Peptides Promote Antioxidant Effect on Caco‐2 Cells.” Food Research International 173: 113291. 10.1016/j.foodres.2023.113291. 37803604

[fsn371806-bib-0025] Dineshbhai, C. K. , B. Basaiawmoit , A. A. Sakure , et al. 2022. “Exploring the Potential of *Lactobacillus* and *Saccharomyces* for Biofunctionalities and the Release of Bioactive Peptides From Whey Protein Fermentate.” Food Bioscience 48: 101758. 10.1016/j.fbio.2022.101758.

[fsn371806-bib-0026] Du, Z. , X. Ding , Y. Xu , and Y. Li . 2023. “UniDL4BioPep: A Universal Deep Learning Architecture for Binary Classification in Peptide Bioactivity.” Briefings in Bioinformatics 24, no. 3: bbad135. 10.1093/bib/bbad135. 37020337

[fsn371806-bib-0027] Elisha, C. , P. Bhagwat , and S. Pillai . 2025. “In Silico and In Vitro Analysis of Dipeptidyl Peptidase‐IV and Angiotensin‐Converting Enzyme Inhibitory Peptides Derived From Milk Lactoferrin.” International Dairy Journal 160, no. 106092: 106092. 10.1016/j.idairyj.2024.106092.

[fsn371806-bib-0028] Gallego, M. , L. Mora , and F. Toldrá . 2024. “Effect of Ultrasound and Enzymatic Pre‐Treatments on the Profile of Bioactive Peptides of Beef Liver Hydrolysates.” Food Research International 197, no. 115240: 115240. 10.1016/j.foodres.2024.115240. 39593322

[fsn371806-bib-0029] García‐Escamilla, A. E. , Z. D. Solís‐Macías , G. M. Rodríguez‐Serrano , et al. 2024. “ *Lacticaseibacillus rhamnosus* GG and * Lactobacillus casei Shirota* Growth on a Medium Enriched With Rye Protein, and Assessment of DPP‐IV Inhibitory Activity.” Biology and Life Sciences Forum 40, no. 1: 34. 10.3390/blsf2024040034.

[fsn371806-bib-0030] Ge, Y. , X. Yu , X. Zhao , et al. 2024. “Fermentation Characteristics and Postacidification of Yogurt by *Streptococcus thermophilus* CICC 6038 and *Lactobacillus delbrueckii* Ssp. *Bulgaricus* CICC 6047 at Optimal Inoculum Ratio.” Journal of Dairy Science 107, no. 1: 123–140. 10.3168/jds.2023-23817. 37641256

[fsn371806-bib-0031] Ghailan, A. Z. , and A. K. Niamah . 2025. “ *Streptococcus thermophilus* : Metabolic Properties, Functional Features, and Useful Applications.” Applied Microbiology 5, no. 4: 101. 10.3390/applmicrobiol5040101.

[fsn371806-bib-0032] González‐Olivares, L. G. , J. Añorve‐Morga , A. Castañeda‐Ovando , E. Contreras‐López , and J. Jaimez‐Ordaz . 2014. “Peptide Separation of Commercial Fermented Milk During Refrigerated Storage.” Food Science and Technology 34, no. 4: 674–679. 10.1590/1678-457x.6415.

[fsn371806-bib-0033] González‐Olivares, L. G. , A. Castañeda‐Ovando , A. S. Granados‐Benitez , E. P. Castañeda‐Ovando , E. Contreras‐López , and J. Jaimez‐Ordaz . 2023. “Metal‐Binding Capacity of Whey‐Natural Peptides: Experimental Study and In Vitro FRAP‐Antioxidant Activity.” Biointerface Research in Applied Chemistry 13, no. 6: 565. 10.33263/BRIAC136.565.

[fsn371806-bib-0034] Guha, S. , H. Sharma , G. K. Deshwal , and P. S. Rao . 2021. “A Comprehensive Review on Bioactive Peptides Derived From Milk and Milk Products of Minor Dairy Species.” Food Production, Processing and Nutrition 3, no. 1: 2. 10.1186/s43014-020-00045-7.

[fsn371806-bib-0035] Guo, H. , C. Zang , L. Zheng , et al. 2024. “Novel Antioxidant Peptides From Fermented Whey Protein by *Lactobacillus rhamnosus* B2‐1: Separation and Identification by In Vitro and In Silico Approaches.” Journal of Agricultural and Food Chemistry 72, no. 42: 23306–23319. 10.1021/acs.jafc.4c07531. 39392363 PMC11505895

[fsn371806-bib-0036] Guzmán, F. , A. Gauna , T. Roman , et al. 2021. “Tea Bags for Fmoc Solid‐Phase Peptide Synthesis: An Example of Circular Economy.” Molecules 26, no. 16: 5035. 10.3390/molecules26165035. 34443624 PMC8399505

[fsn371806-bib-0037] Guzmán‐Rodríguez, F. , L. Gómez‐Ruiz , and A. Cruz‐Guerrero . 2024. “Antithrombotic and Ace‐Inhibitory Activity of Milk Fermented by *Lacticaseibacillus rhamnosus* GG and *Lactobacillus delbrueckii* Ssp. *Bulgaricus* .” International Journal of Food Science and Technology 59, no. 12: 9417–9424. 10.1111/ijfs.17589.

[fsn371806-bib-0038] Helal, A. , C. Nasuti , L. Sola , G. Sassi , D. Tagliazucchi , and L. Solieri . 2023. “Impact of Spontaneous Fermentation and Inoculum With Natural Whey Starter on PEPTIDOMIC Profile and Biological Activities of Cheese Whey: A Comparative Study.” Fermentation 9, no. 3: 270. 10.3390/fermentation9030270.

[fsn371806-bib-0039] Helal, A. , S. Pierri , D. Tagliazucchi , and L. Solieri . 2023. “Effect of Fermentation With *Streptococcus thermophilus* Strains on In Vitro Gastro‐Intestinal Digestion of Whey Protein Concentrates.” Microorganisms 11, no. 7: 1742. 10.3390/microorganisms11071742. 37512914 PMC10386367

[fsn371806-bib-0040] Hernández‐Riveros, E. , L. B. Olvera‐Rosales , J. Jaimez‐Ordaz , et al. 2024. “Production of an Ice Cream Base With Added *Lacticaseibacillus rhamnosus* GG and Aguamiel Syrup: Probiotic Viability and Antihypertensive Capacity.” Dairy 5, no. 3: 451–463. 10.3390/dairy5030035.

[fsn371806-bib-0041] Hetta, H. F. , N. Sirag , S. M. Alsharif , et al. 2024. “Antimicrobial Peptides: The Game‐Changer in the Epic Battle Against Multidrug‐Resistant Bacteria.” Pharmaceuticals 17, no. 11: 1555. 10.3390/ph17111555. 39598464 PMC11597525

[fsn371806-bib-0042] Heydari, S. , S. E. Hosseini , A. M. Mortazavian , and S. Taheri . 2023. “Extraction of Bioactive Peptides Produced in Probiotic Yoghurt and Determination of Their Biological Activities.” International Dairy Journal 139: 105544. 10.1016/j.idairyj.2022.105544.

[fsn371806-bib-0043] Hou, H. , S. Wang , X. Zhu , et al. 2018. “A Novel Calcium‐Binding Peptide From Antarctic Krill Protein Hydrolysates and Identification of Binding Sites of Calcium‐Peptide Complex.” Food Chemistry 243: 389–395. 10.1016/j.foodchem.2017.09.152. 29146354

[fsn371806-bib-0044] Hrynkiewicz, M. , A. Iwaniak , P. Minkiewicz , M. Darewicz , and W. Płonka . 2023. “Analysis of Structure–Activity Relationships of Food‐Derived DPP IV Inhibitory di‐ and Tripeptides Using Interpretable Descriptors.” Applied Sciences 13, no. 23: 12935. 10.3390/app132312935.

[fsn371806-bib-0081] Hussein, F. A. , S. Y. Chay , M. Zarei , et al. 2020. “Whey Protein Concentrate as a Novel Source of Bifunctional Peptides with Angiotensin‐I Converting Enzyme Inhibitory and Antioxidant Properties: RSM Study.” Food 9, no. 1: 64. 10.3390/foods9010064. PMC7022510 31936191

[fsn371806-bib-0045] Innocente, N. , S. Calligaris , G. Di Filippo , S. Melchior , M. Marino , and M. C. Nicoli . 2023. “Process Design for the Production of Peptides From Whey Protein Isolate With Targeted Antimicrobial Functionality.” International Journal of Food Science & Technology 58, no. 5: 2505–2517. 10.1111/ijfs.16393.

[fsn371806-bib-0046] Jia, C. , N. Hussain , O. Joy Ujiroghene , X. Pang , S. Zhang , and J. Lu . 2020. “Generation and Characterization of Dipeptidyl Peptidase‐IV Inhibitory Peptides From Trypsin‐Hydrolyzed α‐Lactalbumin‐Rich Whey Proteins.” Food Chemistry 318: 126333. 10.1016/j.foodchem.2020.126333. 32151919

[fsn371806-bib-0047] Jiang, Y. , S. Li , L. Jiang , G. Mu , and S. Jiang . 2025. “Immunomodulatory Activity and Molecular Mechanisms of Action of Peptides Derived From Casein Hydrolysate by Alcalase and Flavourzyme Based on Virtual Screening.” Journal of Dairy Science 108, no. 3: 2152–2168. 10.3168/jds.2024-25224. 39603497

[fsn371806-bib-0048] Jimenez, R. , D. Yurk , S. Dell , et al. 2024. “Resonance Sonomanometry for Noninvasive, Continuous Monitoring of Blood Pressure.” PNAS Nexus 3, no. 7: gae252. 10.1093/pnasnexus/pgae252. PMC11287871 39081785

[fsn371806-bib-0049] Kapil, S. , and V. Sharma . 2021. “Amino Acids in Antimicrobial Peptides: A Potential Approach to Treat and Combat Antimicrobial Resistance.” Canadian Journal of Microbiology 67: 119–137. 32783775 10.1139/cjm-2020-0142

[fsn371806-bib-0050] Kotsaki, P. , M. Aspri , and P. Papademas . 2025. “Novel Whey Fermented Beverage Enriched With a Mixture of Juice Concentrates: Evaluation of Antimicrobial, Antioxidant, and Angiotensin‐I Converting Enzyme Inhibitory (ACE) Activities Before and After Simulated Gastrointestinal Digestion.” Microorganisms 13, no. 7: 1490. 10.3390/microorganisms13071490. 40732000 PMC12298918

[fsn371806-bib-0051] Lemes, A. C. , J. G. de Oliveira Filho , S. S. Fernandes , G. V. Gautério , and M. B. Egea . 2023. “Bioactive Peptides From Protein‐Rich Waste.” In Agricultural Waste: Environmental Impact, Useful Metabolites and Energy Production, 139–166. Springer.

[fsn371806-bib-0052] Liu, M. , Z. Li , Q. Chen , et al. 2024. “Preparation and Characterization of Grouper Bone Peptides‐Calcium Complex by Lactic Acid Bacteria Fermentation.” LWT 201: 116224. 10.1016/j.lwt.2024.116224.

[fsn371806-bib-0053] Mazorra‐Manzano, M. A. , A. Martínez‐García , M. J. Torres‐Llanez , et al. 2025. “Techno‐Functional Properties of Mexican Cheese Whey Requesón Powder: Effects of Air‐Convective Drying and Natural Gum Addition.” Dairy 6, no. 4: 32. 10.3390/dairy6040032.

[fsn371806-bib-0054] Mazorra‐Manzano, M. A. , G. R. Robles‐Porchas , D. A. González‐Velázquez , et al. 2020. “Cheese Whey Fermentation by Its Native Microbiota: Proteolysis and Bioactive Peptides Release With ACE‐Inhibitory Activity.” Fermentation 6, no. 1: 19. 10.3390/fermentation6010019.

[fsn371806-bib-0055] Olvera‐Rosales, L. B. , A. E. Cruz‐Guerrero , J. Jaimez‐Ordaz , et al. 2023. “Differences in the Proteolytic System of Lactic Acid Bacteria Affect the Release of DPP‐IV Inhibitory Peptides From Whey Proteins.” Dairy 4, no. 3: 515–526. 10.3390/dairy4030035.

[fsn371806-bib-0056] Olvera‐Rosales, L. B. , E. Pérez‐Escalante , A. Castañeda‐Ovando , et al. 2023. “ACE‐Inhibitory Activity of Whey Proteins Fractions Derived of Fermentation by *Lacticaseibacillus rhamnosus* GG and *Streptococcus thermophilus* SY‐102.” Food 12, no. 12: 2416. 10.3390/foods12122416. PMC10297981 37372627

[fsn371806-bib-0057] Parra‐Ocampo, K. A. , S. T. Martín‐del‐Campo , J. G. Montejano‐Gaitán , R. Zárraga‐Alcántar , and A. Cardador‐Martínez . 2020. “Evaluation of Biological, Textural, and Physicochemical Parameters of Panela Cheese Added With Probiotics.” Food 9, no. 10: 1507. 10.3390/foods9101507. PMC7589322 33096619

[fsn371806-bib-0058] Perlikowska, R. , J. Silva , C. Alves , P. Susano , and R. Pedrosa . 2022. “The Therapeutic Potential of Naturally Occurring Peptides in Counteracting SH‐SY5Y Cells Injury.” International Journal of Molecular Sciences 23, no. 19: 11778. 10.3390/ijms231911778. 36233079 PMC9569762

[fsn371806-bib-0059] Ramírez‐Rivas, I. K. , N. Gutiérrez‐Méndez , A. L. Rentería‐Monterrubio , et al. 2022. “Effect of Packaging and Salt Content and Type on Antioxidant and ACE‐Inhibitory Activities in Requeson Cheese.” Food 11, no. 9: 1264. 10.3390/foods11091264. PMC9102267 35563990

[fsn371806-bib-0060] Rodríguez‐Serrano, G. M. , J. M. García‐Garibay , A. E. Cruz‐Guerrero , et al. 2018. “Proteolytic System of *Streptococcus thermophilus* .” Journal of Microbiology and Biotechnology 28, no. 10: 1581–1588. 10.4014/jmb.1807.07017. 30196594

[fsn371806-bib-0061] Sato, K. , S. Miyasaka , A. Tsuji , and H. Tachi . 2018. “Isolation and Characterization of Peptides With Dipeptidyl Peptidase IV (DPPIV) Inhibitory Activity From Natto Using DPPIV From *Aspergillus oryzae* .” Food Chemistry 261: 51–56. 10.1016/j.foodchem.2018.04.029. 29739605

[fsn371806-bib-0062] Sebastián‐Nicolás, J. L. , E. Contreras‐López , J. G. Pérez‐Flores , et al. 2024. “Improvement of the Antioxidant Capacity of a Yogurt Enriched With Aqueous Ginger Extract ( *Zingiber officinale* ).” Revista Mexicana de Ingenieria Quimica 23, no. 3: Bio24254. 10.24275/rmiq/Bio24254.

[fsn371806-bib-0063] Sebastián‐Nicolas, J. L. , E. Contreras‐López , J. Ramírez‐Godínez , et al. 2021. “Milk Fermentation by *Lacticaseibacillus rhamnosus* GG and *Streptococcus thermophilus* SY‐102: Proteolytic Profile and ACE‐Inhibitory Activity.” Fermentation 7, no. 4: 215. 10.3390/fermentation7040215.

[fsn371806-bib-0064] Sebastián‐Nicolás, J. L. , L. G. González‐Olivares , G. A. Vázquez‐Rodríguez , C. Lucho‐Constatino , A. Castañeda‐Ovando , and A. E. Cruz‐Guerrero . 2020. “Valorization of Whey Using a Biorefinery.” Biofuels, Bioproducts and Biorefining 14, no. 5: 1010–1027. 10.1002/bbb.2100.

[fsn371806-bib-0065] Solieri, L. , M. Valentini , A. Cattivelli , et al. 2022. “Fermentation of Whey Protein Concentrate by *Streptococcus thermophilus* Strains Releases Peptides With Biological Activities.” Process Biochemistry 121: 590–600. 10.1016/j.procbio.2022.08.003.

[fsn371806-bib-0066] Song, Y. , J. Zhao , W. Liu , et al. 2021. “Exploring the Industrial Potential of *Lactobacillus delbrueckii* Ssp. *Bulgaricus* by Population Genomics and Genome‐Wide Association Study Analysis.” Journal of Dairy Science 104, no. 4: 4044–4055. 10.3168/jds.2020-19467. 33663860

[fsn371806-bib-0067] Srivastava, U. , B. H. Nataraj , M. Kumari , et al. 2022. “Antioxidant and Immunomodulatory Potency of *Lacticaseibacillus rhamnosus* NCDC24 Fermented Milk‐Derived Peptides: A Computationally Guided In‐Vitro and Ex‐Vivo Investigation.” Peptides 155: 170843. 10.1016/j.peptides.2022.170843. 35878657

[fsn371806-bib-0068] Szymoniak, L. , D. Claveau‐Mallet , M. Haddad , and B. Barbeau . 2022. “Application of Magnesium Oxide Media for Remineralization and Removal of Divalent Metals in Drinking Water Treatment: A Review.” Water 14, no. 4: 633. 10.3390/w14040633.

[fsn371806-bib-0069] Talapko, J. , T. Meštrović , M. Juzbašić , et al. 2022. “Antimicrobial Peptides‐Mechanisms of Action, Antimicrobial Effects and Clinical Applications.” Antibiotics 11, no. 10: 1417. 10.3390/antibiotics11101417. 36290075 PMC9598582

[fsn371806-bib-0070] Ungureanu‐Rusu, M. C. , A. Rusu , and A. Păucean . 2025. “Whey—A Valuable Technological Resource for the Production of New Functional Products With Added Health‐Promoting Properties.” Discover Food 5, no. 1: 12. 10.1007/s44187-025-00430-6. PMC12732941 41464965

[fsn371806-bib-0071] Wang, L. , Y. Ding , X. Zhang , et al. 2018. “Isolation of a Novel Calcium‐Binding Peptide From Wheat Germ Protein Hydrolysates and the Prediction for Its Mechanism of Combination.” Food Chemistry 239: 416–426. 10.1016/j.foodchem.2017.06.090. 28873586

[fsn371806-bib-0072] Worsztynowicz, P. , W. Białas , and W. Grajek . 2020. “Integrated Approach for Obtaining Bioactive Peptides From Whey Proteins Hydrolyzed Using a New Proteolytic Lactic Acid Bacteria.” Food Chemistry 312: 126035. 10.1016/j.foodchem.2019.126035. 31901822

[fsn371806-bib-0073] Wu, T. , S. Guo , L.‐Y. Kwok , H. Zhang , and J. Wang . 2025. “Temperature‐Dependent Metabolic Interactions Between *Streptococcus thermophilus* and *Lactobacillus delbrueckii* Ssp. *Bulgaricus* in Milk Fermentation: Insights From Gas Chromatography–Ion Mobility Spectrometry Metabolomics.” Journal of Dairy Science 108, no. 1: 242–256. 10.3168/jds.2024-25153. 39343235

[fsn371806-bib-0074] Wu, Y. , Y. Wang , Z. Ma , G. Mu , and F. Qian . 2024. “Novel Insights Into Whey Protein Peptide‐Iron Chelating Agents: Structural Characterization, In Vitro Stability and Functional Properties.” Food Bioscience 60: 104317. 10.1016/j.fbio.2024.104317.

[fsn371806-bib-0075] Yao, X. , X. Cao , L. Chen , and W. Liao . 2024. “Research Progress of Food‐Derived Antihypertensive Peptides in Regulating the Key Factors of the Renin–Angiotensin System.” Nutrients 17, no. 1: 97. 10.3390/nu17010097. 39796531 PMC11722916

[fsn371806-bib-0076] Zaresharifi, S. , M. Niroomand , S. Borran , and S. Dadkhahfar . 2024. “Dermatological Side Effects of Dipeptidyl Peptidase‐4 Inhibitors in Diabetes Management: A Comprehensive Review.” Clinical Diabetes and Endocrinology 10, no. 1: 6. 10.1186/s40842-024-00165-w. 38523307 PMC10962164

[fsn371806-bib-0077] Zhang, C. , B. Du , Z. Song , et al. 2023. “Antioxidant Activity Analysis of Collagen Peptide‐Magnesium Chelate.” Polymer Testing 117: 107822. 10.1016/j.polymertesting.2022.107822.

[fsn371806-bib-0078] Zhang, M. , L. Zhu , H. Zhang , et al. 2023. “Identification of Novel Dipeptidyl Peptidase‐4 Inhibitory Peptides From PEA Proteins: A Combined In Silico and In Vitro Study.” Food Bioscience 56: 103374. 10.1016/j.fbio.2023.103374.

[fsn371806-bib-0079] Zhang, Y. , X. Ding , and M. Li . 2021. “Preparation, Characterization and *in Vitro* Stability of Iron‐Chelating Peptides From Mung Beans.” Food Chemistry 349: 129101. 10.1016/j.foodchem.2021.129101. 33540219

[fsn371806-bib-0080] Zhou, L. , C. Xiao , J. Gao , et al. 2024. “Preparation and Identification of Novel DPP‐IV Inhibitory Peptides From *Musculus senhousei* : Peptidomic Analysis, Molecular Simulation, and Validation.” Food Bioscience 59: 103832. 10.1016/j.fbio.2024.103832.

